# A retrospective comparative study of postoperative sagittal balance in isthmic L5–S1 spondylolisthesis using single segment or two-segment pedicle screw fixation

**DOI:** 10.1186/s12891-022-05098-y

**Published:** 2022-02-12

**Authors:** Xiaofeng Shao, Hao Liu, Jian Wu, Zhonglai Qian, Rui Qu, Tao Liu

**Affiliations:** 1 Department of Orthopedic Surgery, The First Affiliated Hospital of Soochow University, No. 188 Shizi Street, Suzhou, Jiangsu 215006 P.R. China; 2 The Department of Orthopedic Surgery, Changsu No.2. People’s Hospital, Jiangsu, Suzhou, China; 3 Department of Orthopedic Surgery, The Affiliated Suzhou Hospital of Nanjing Medical University, No. 26 Daoqian Street, Suzhou, Jiangsu 215006 P.R. China

**Keywords:** Lumbar spondylolisthesis, Sagittal balance, Lumbar pedicle screw fixation

## Abstract

**Objective:**

To compare the radiographic parameters and clinical outcomes of isthmic L5–S1 spondylolisthesis with single segment or two-segment pedicle screw fixation.

**Methods:**

Between January 2018 and January 2019, a total of 76 patients with isthmic L5–S1 spondylolisthesis were included in this study. All patients were treated with varying numbers of pedicle screw fixation with single-segment fusion during posterior lumbar interbody fusion (PLIF). Patients were divided into two groups, based on the number of pedicle screws placed during fixation, namely, 4 screws (4S) group and 6 screws (6S) group. Subsequently, the sagittal balance parameters were measured, which included slippage degree (SD), lumbar lordosis (LL), segmental lordosis (SL), pelvic incidence (PI), pelvic tilt (PT), sacral slope (SS), and sagittal vertical axis (SVA). Clinical functional outcomes were assessed using the visual analog scale (VAS) for back pain and the oswestry disability index (ODI) scores.

**Results:**

The 4S group comprised of 10 males and 27 females, with a median age of 55.2 ± 10.8 years old and a mean follow-up of 16.95 ± 4.16 months. The 6S group comprised of 14 males and 25 females, with a median age of 58.1 ± 7.5 years old and a median follow-up of 17.33 ± 3.81 months. No significant differences were evident in all preoperative parameters between both groups. In contrast, the postoperative LL, SL, PT, SS, and SD values increased significantly, compared to the preoperative values in both groups (all *P* < 0.05). At the last follow-up, the 6S group exhibited better correction in LL, SL, and PT, relative to the 4S group (all *P* < 0.05). A significant SD difference was observed between both groups at all points post surgery (*P* < 0.05). The postoperative slip correction rate was significantly larger in the 6S group, compared to the 4S group (*P* < 0.05). The postoperative VAS and ODI scores of both groups improved significantly, when compared to the preoperative scores (both *P* < 0.05). However, there were no significant differences in the ODI and VAS scores between the two groups at all time points.

**Conclusions:**

The clinical outcomes of both approaches appeared to be satisfactory. In terms of short-term outcomes, the 6S group exhibited better spinal sagittal restoration and stability than the 4S group.

## Introduction

Isthmic spondylolisthesis is a common spinal disease that results from a pars interarticular defect and the L5–S1 region is the most commonly affected region. Lumbar spondylolisthesis manifestations are related to lumbar spinal stenosis, which produces symptomatic compression of the neural element. However, it can also surface due to segmental instability. This condition is initially treated with conservative management strategies. But, in case a patient fails to respond, surgery is recommended. Surgical intervention can effectively relieve dural sac and nerve root compression, prevent spondylolisthesis degeneration from aggravation, correct spinal deformity, and stabilize the lumbar spine [1]. Posterior lumbar interbody fusion (PLIF) is a widely used surgical approach for treating lumbar spondylolisthesis. Moreover, pedicle screw placement is crucial to the success of PLIF-based fusion, and has shown remarkable clinical outcomes [2, 3].

In recent years, spinopelvic sagittal balance is widely recognized as a crucial factor regulating clinical outcomes in patients undergoing spinal surgery [4] . Since lumbar spondylolisthesis often combines with other deformities like forward slip and kyphosis, it often results in a global sagittal imbalance of the spine. Spinopelvic sagittal balance is essential in the evaluation and treatment of patients with spondylolisthesis. Traditionally, fixation placement involves inserting two pedicle screws in the slipped and lower vertebral bodies, in an approach known as short segment fixation. In our study, additional pedicle screws were implanted into the upper vertebrae of the slipped vertebrae, thereby making it a long segment fixation. Till date, limited studies reported on the influence of short versus long segment pedicle screw fixation on sagittal balance in lumbar spondylolisthesis. Given its significance in determining surgical success, it is essential to examine the outcomes of inserting varying number of screws on sagittal balance in patients with lumbar spondylolisthesis.

In this retrospective study, we compared the clinical functional outcomes and sagittal balance parameters of patients with isthmic spondylolisthesis treated with varying number of screw implants during PLIF surgery.

## Materials and methods

### Patient population

Between January 2018 and January 2019, 76 patients with L5-S1 isthmic spondylolisthesis, who underwent PLIF surgery, were selected for this study. The patients were divided into two groups, based on the surgical methods: Four screws group (4S group): 37 patients received a single fixation with four pedicle screws. Six screws group (6S group): 39 patients received a single fixation with six pedicle screws. We obtained ethical approval from the Medical Ethics Committee of the First Affiliated Hospital of Soochow University prior to the initiation of this study.

### Inclusion and exclusion criteria

The inclusion criteria were as follows: (1) patients with L5-S1 isthmic spondylolisthesis; (2) patients experiencing symptoms of unilateral/double sciatica or intermittent claudication, with low back pain, who are unresponsive to conservative therapy for over 6 months; (3) patients treated with varying numbers of pedicle screws during the L5–S1 fusion PLIF surgery; (4) Patient follow-up duration of more than 1 year. The exclusion criteria were as follows: (1) intervertebral space infection, spinal tumors, congenital spinal deformity, and acute vertebral fractures; (2) patients with no valid follow-up information.

### Surgical procedure

All surgeries were conducted by the same two highly experienced orthopedic surgeons in order to minimize influence of varying surgical techniques. A posterior median incision was made to expose the spinous process.

**Fig. 1:**
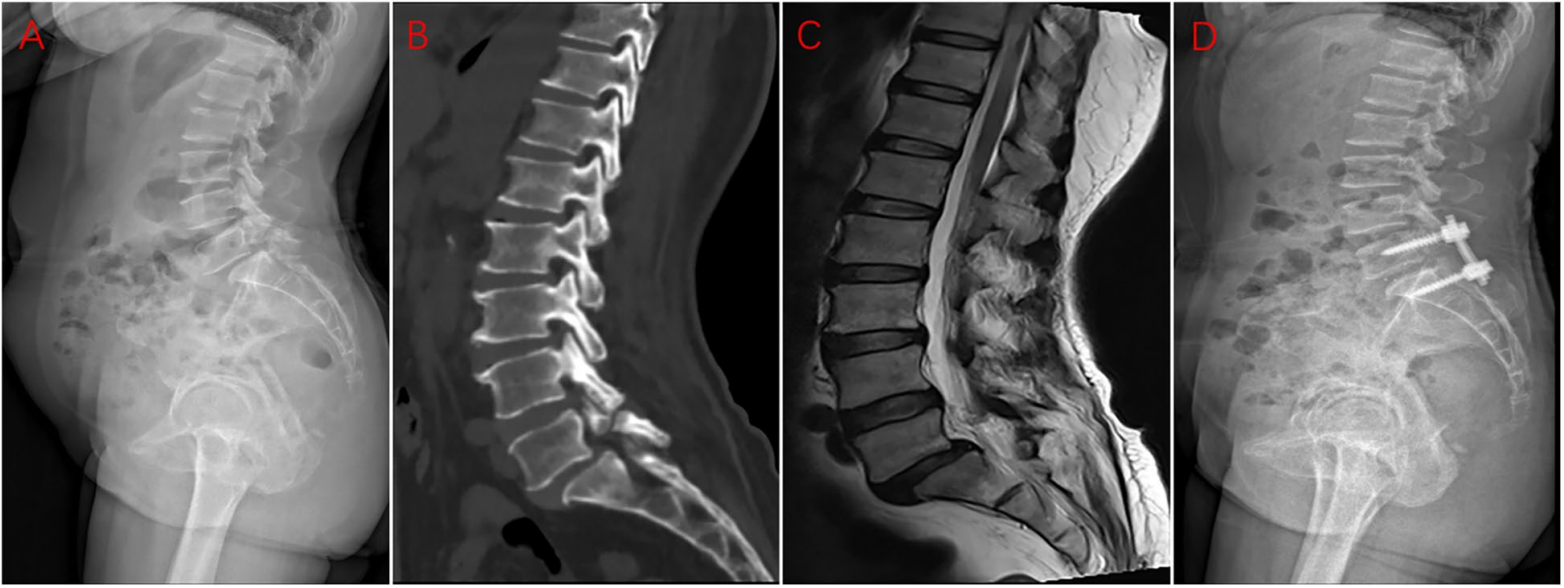
The preoperative lateral X-ray image (**a**), preoperative sagittal CT image (**b**), preoperative sagittal MRI image (**c**), and postoperative (**d**) lateral X-ray image of a 60-year-old female patient, who underwent L5-S1 PLIF, with 4 screws to correct lumbar spondylolisthesis

In the 4S group, the L5 vertebra was conventionally implanted with two pedicle screws, and two additional pedicle screws were inserted into the S1 vertebra, all under the guidance of C-arm X-ray fluoroscopy (Fig. 1). Next, we performed bilateral laminotomies and thorough discectomy prior to the placement of a properly sized cage, packed with laminectomy bone. Upon completion of lifting and reduction, titanium rods were placed and the nut was locked. The PLIF was limited to L5–S1.

**Fig. 2:**
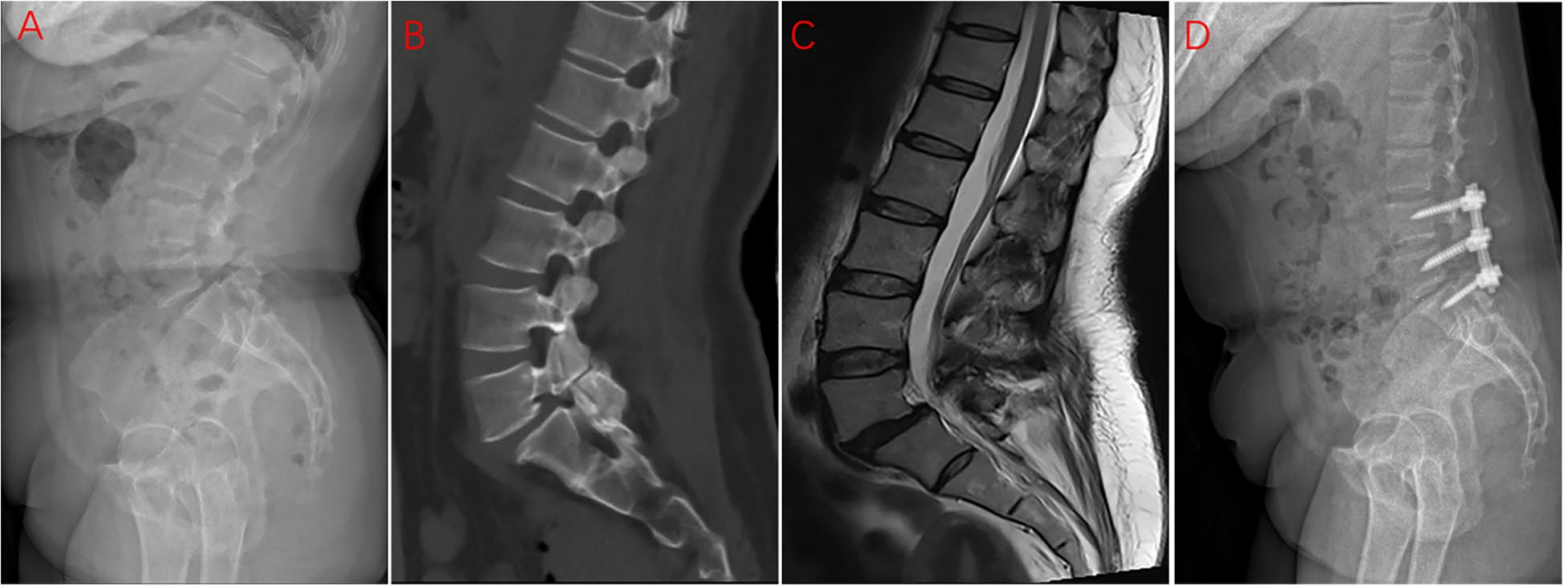
The preoperative lateral X-ray image (**a**), preoperative sagittal CT image (**b**), preoperative sagittal MRI image (**c**), and postoperative (**d**) lateral X-ray image of a 63-year-old female patient, who underwent L5-S1 PLIF, with 6 screws to correct lumbar spondylolisthesis

In the 6S group, the L5 vertebra was conventionally implanted with two pedicle screws, the S1 vertebra was fixed with two pedicle screws, and the L4 vertebra was implanted with two additional pedicle screws (Fig. 2). The incision and surgical procedure of the laminectomy and associated decompression followed the same protocol as the 4S group. Using the cranial and caudal vertebral bodies of the slipped vertebra as anchors and a middle screw as the leverage point, we performed middle bridge-shaped pulling reduction. Finally, the titanium rods were placed and the nut was locked. The intervertebral fusion was limited to L5–S1 and the posterior fixation was limited to L4–S1.

### Radiographic parameters

Alterations in lumbar lordosis (LL), slippage degree (SD), segmental lordosis (SL), pelvic incidence (PI), sacral slope (SS), pelvic tilt (PT), and sagittal vertical axis (SVA) were assessed by the EOS system. Two observations were made, at least two weeks apart, by two independent spinal surgeons, and the mean radiological spinopelvic parameter values were used in subsequent analysis.

**Fig. 3:**
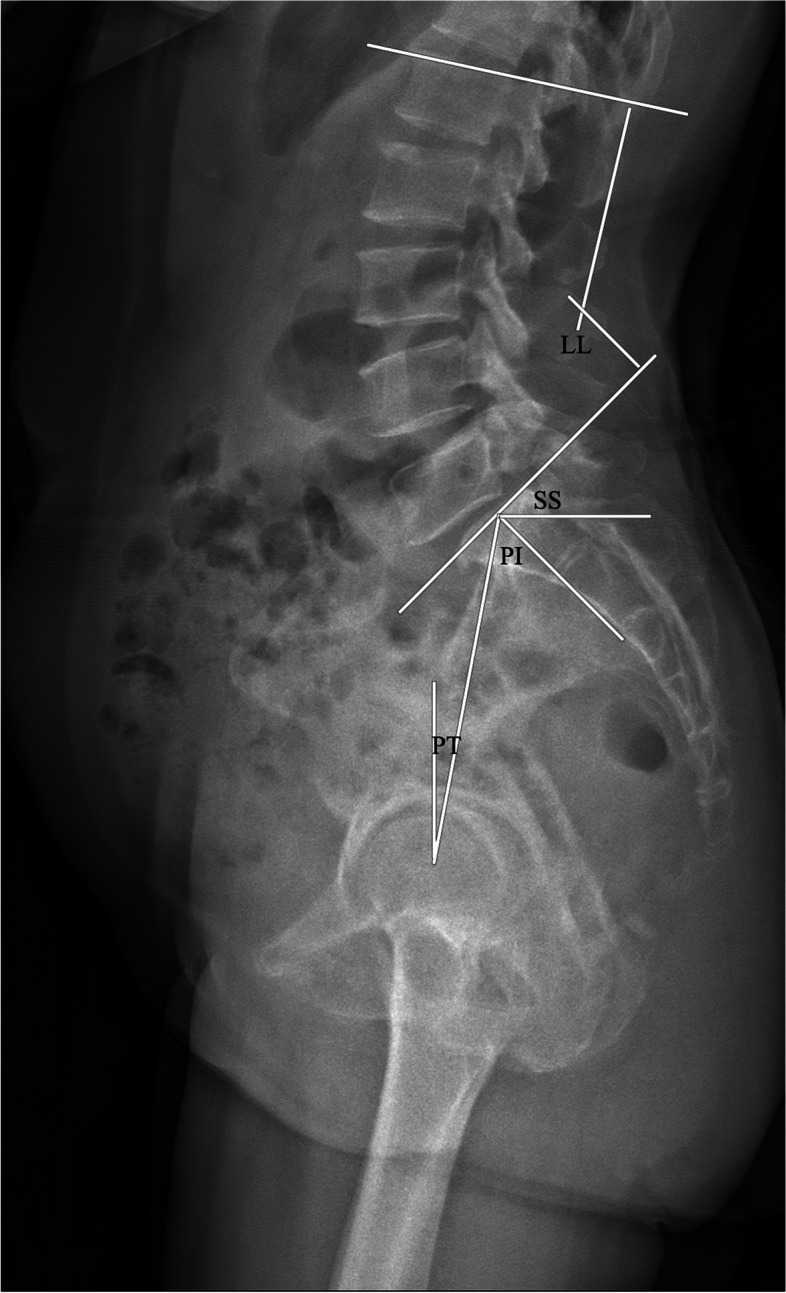
The radiological parameter measurements (LL, PI, PT, and SS)

**Fig. 4:**
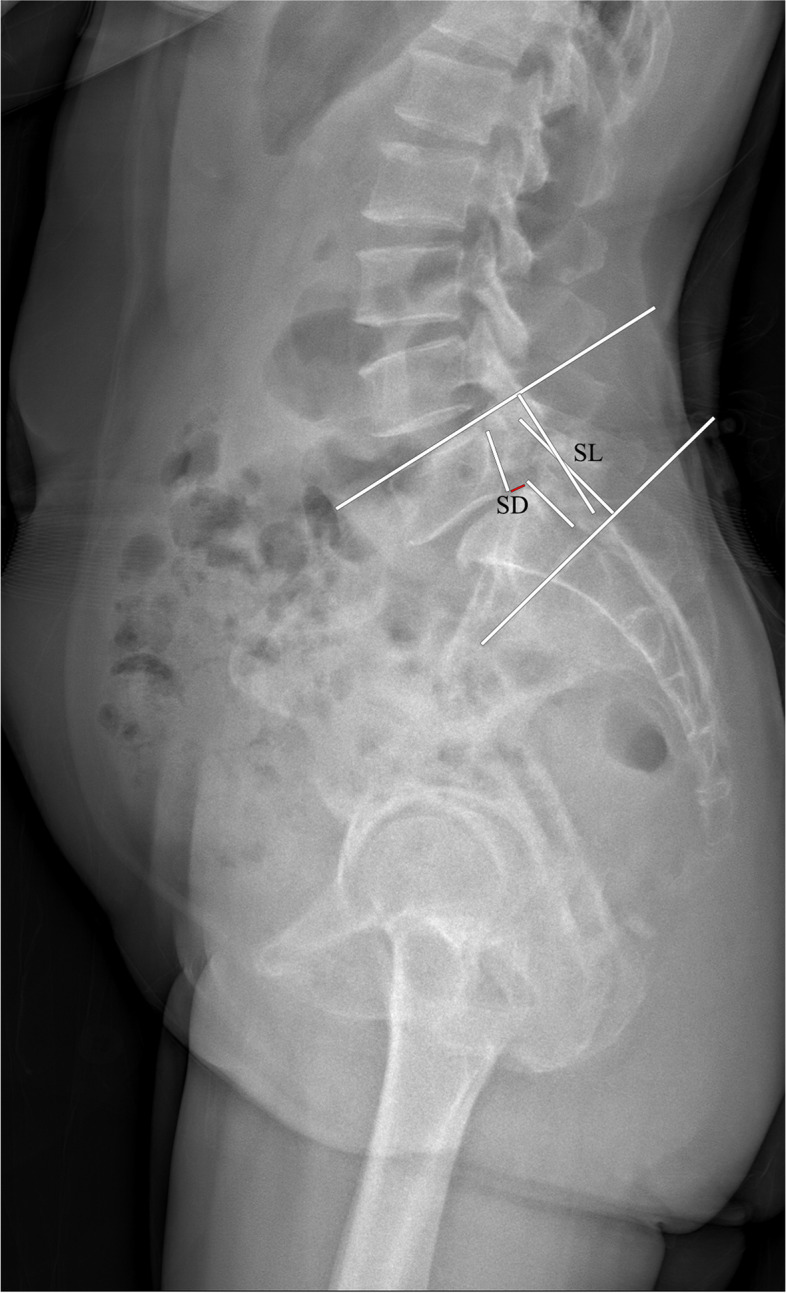
The radiological parameter measurements (SD and LL)

**Fig. 5:**
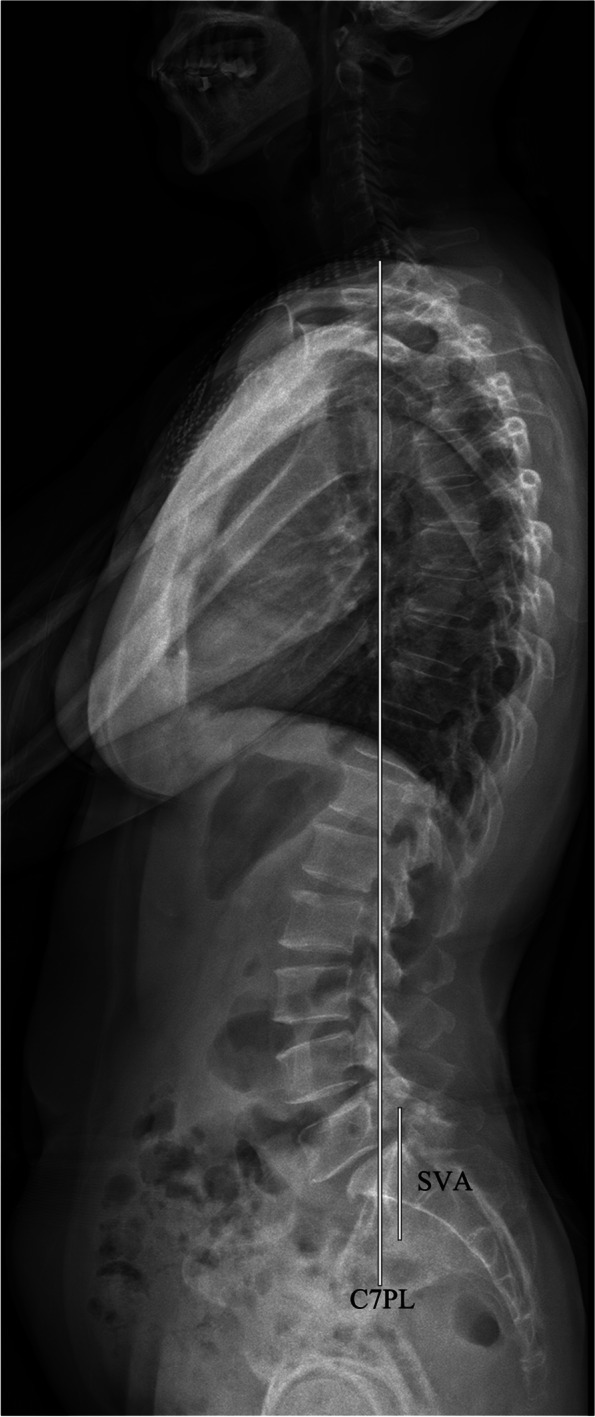
The radiological SVA measurement

LL was defined as the angle between the upper endplate of the L1 and S1 (Fig. 3). PI was the angle between the line perpendicular to the midpoint of the S1 upper endplate and the line connecting the midpoint of S1 upper endplate to the bicoxo-femoral axis. PT was the angle between the vertical line and the line connecting the midpoint of the S1 upper endplate to the bicoxo-femoral axis. SS was the angle between the horizontal line and the upper endplate of S1. SL was the angle between the upper endplate of L5 and the lower endplate of the S1 vertebra. SD was assessed by the Meyerding grade (Fig. 4). The slip correction rate was calculated as follows: [(preoperative slip percentage − postoperative slip percentage) / (preoperative slip percentage)] × 100. SVA was the distance between the posterosuperior corner of S1 and the C7 plumb line (Fig. 5).

### Clinical outcomes

The visual analog scale (VAS) score was used to assess back pain in patients; lower scores indicated less pain. The oswestry disability index (ODI) was employed to evaluate quality of patient life; a higher score indicated worsening quality of life.

### Statistical analysis

The SPSS 19.0 software (IBM, Armonk, NY, USA) was used for all data analyses. Data are presented as mean ± standard. The student’s t test was used for numerical data, including, age, BMI, BMD, radiographic parameters, VAS scores, and ODI in both groups. The Pearson’s Chi-square and Fisher exact tests were used to analyze categorical data, including, gender and slip grade. A *p* value less than 0.05 was considered statistically significant.

## Results

### Demographic

The 4S group consisted of 10 males and 27 females, with a mean age of 55.2 ± 10.8 years old. All 37 adult patients underwent a single-segment fusion with 4 screw fixations (4S group) and a median follow-up of 16.95 ± 4.16 months. The 6S group consisted of 14 males and 25 females, with a median age of 58.1 ± 7.5 years old. No significant differences were observed in gender, age, BMI, BMD, slip grade, and preoperative SVA. The basic patient characteristics are presented in Table 1.

**Table 1:** Demographic data of patients in the two groups

	4S group	6S group	*P* value
Number	37	39	
Gender(male/female)	10/27	14/25	0.405
Age (years)	55.2 ± 10.8	58.1 ± 7.5	0.176
BMI (kg/m2)	24.73 ± 2.94	25.14 ± 3.79	0.601
BMD(T-score)	−1.95 ± 1.05	−2.01 ± 0.79	0.778
Meyerding grade	I° (*n* = 24)	I° (*n* = 24)	0.764
	II° (*n* = 13)	II° (*n* = 15)	
SVA (%)			
> 2 cm but ≤5 cm	67.6%(*n =* 25)	64.1%(*n* = 25)	0.750
> 5 cm but ≤10 cm	32.4%(*n =* 12)	35.9%(*n =* 14)	
Follow-up (months))	16.95 ± 4.16	17.33 ± 3.81	0.679
Operative time (min)	158.03 ± 27.19	187.10 ± 37.41	**0.000**
Blood loss (ml)	166.76 ± 57.54	214.87 ± 75.67	**0.003**
Total cost (USD)	10,670 ± 1658	12,054 ± 1967	**0.028**

*BMI* Body mass index, *BMD* Bone mineral density, *SVA* Sagittal vertical axis

Bold represents there is statistical significance between the groups, *p* < 0.05

### Radiological outcomes

The LL, SL, PT, SS, and SD increased significantly after surgery, relative to before surgery in both groups (all *P* < 0.05). At the final follow-up, the 6S group exhibited better correction in LL, SL, and PT, compared to the 4S group (all *P* < 0.05). Additionally, the SD improvement was significantly better in the 6S group, compared to the 4S group at all points after surgery (*P* < 0.05). Likewise, the slip correction rate was significantly larger after surgery in the 6S group versus 4S group (*P* < 0.05). No significant preoperative differences were observed in all parameters between the two groups. All radiographic outcomes are summarized in Table 2.

**Table 2:** Comparison of radiographic parameters between the two groups

	4S group	6S group	*P* value
LL (°)			
Pre	52.30 ± 7.94	53.02 ± 8.55	0.705
1 month	57.49 ± 6.51*	60.17 ± 7.10*	0.090
6 months	57.30 ± 6.45*	60.08 ± 7.06*	0.077
Final	56.35 ± 6.15*	59.55 ± 6.84*	**0.036**
SL (°)			
pre	16.32 ± 4.83	16.04 ± 4.54	0.999
1 month	24.37 ± 2.68*	25.21 ± 2.95*	0.197
6 months	24.26 ± 2.60*	25.16 ± 2.91*	0.160
Final	23.49 ± 2.34*	24.88 ± 2.85*	**0.023**
PI (°)			
Pre	61.84 ± 9.35	62.64 ± 10.03	0.721
1 month	62.30 ± 9.02	62.44 ± 9.83	0.949
6 months	62.35 ± 9.07	62.33 ± 9.91	0.992
Final	62.22 ± 9.04	62.41 ± 9.86	0.931
PT (°)			
Pre	21.27 ± 4.00	20.85 ± 5.05	0.690
1 month	16.41 ± 4.23*	15.03 ± 4.27*	0.161
6 months	16.62 ± 4.04*	15.21 ± 4.11*	0.136
Final	17.35 ± 3.98*	15.49 ± 4.05*	**0.047**
SS (°)			
Pre	40.76 ± 10.19	41.87 ± 10.18	0.532
1 month	46.51 ± 9.71*	47.46 ± 10.37*	0.681
6 months	46.11 ± 9.48*	47.15 ± 10.35*	0.650
Final	45.27 ± 9.26*	46.85 ± 10.34*	0.486
SVA (mm)			
Pre	54.43 ± 46.21	56.52 ± 45.76	0.844
1 month	31.26 ± 43.78*	27.81 ± 42.98*	0.730
6 months	32.98 ± 43.85*	28.01 ± 43.07*	0.620
Final	33.71 ± 44.64*	28.98 ± 44.12*	0.644
SD (%)			
Pre	22.40 ± 7.20	23.18 ± 7.53	0.646
1 month	6.67 ± 2.88*	5.23 ± 2.12*	**0.015**
6 months	7.03 ± 2.54*	5.59 ± 1.89*	**0.009**
Final	8.08 ± 2.53*	6.08 ± 2.07*	**0.003**
Slip correction rate (%)	68.86 ± 15.67	77.25 ± 7.4	**0.004**

*LL* Lumbar lordosis, *SL* Segmental lordosis, *PI* Pelvic incidence, *PT* Pelvic tilt, *SS* Sacral slope, *SD* Slippage degree, *SVA* Sagittal vertical axis

Slip correction rate (%) = [(preoperative slip percentage – postoperative slip percentage)/ (preoperative slip percentage) × 100%]

1 month: one month after surgery, 6 months: six months after surgery, FINAL: final follow-up

*Statistically significant compared with the preoperative value, *p* < 0.05

Bold represents there is statistical significance between the groups, *p* < 0.05

### Clinical functional outcomes

In both groups, compared to the preoperative values, the VAS and ODI scores showed significant differences at all points after surgery (all *P* < 0.05). The preoperative VAS and ODI scores in both groups were similar (all *P* < 0.05) and no significant differences were seen in the VAS and ODI scores in both groups at all time points after surgery. All the clinical outcomes are summarized in Table 3.

**Table 3:** Comparison of functional outcomes between the two groups

	4S group	6S group	P value
VAS			
Pre	7.62 ± 1.28	7.46 ± 1.05	0.552
1 month	3.24 ± 0.72*	3.56 ± 0.75*	0.062
6 months	2.46 ± 0.73*	2.38 ± 0.88*	0.668
Final	1.65 ± 0.68*	1.54 ± 0.60*	0.456
ODI			
Pre	54.32 ± 9.55	55.64 ± 10.50	0.569
1 month	26.81 ± 5.88*	27.41 ± 5.96*	0.660
6 months	20.65 ± 3.43*	19.08 ± 4.49*	0.092
Final	18.86 ± 4.77*	18.23 ± 4.15*	0.540
Chronic low back pain	2	1	0.525

*VAS* Visual analogue scale, *ODI* Oswestry Disability Index, *CLBP* Chronic low back pain

1 month: one month after surgery, 6 months: six months after surgery, FINAL: final follow-up

*Statistically significant compared with the preoperative value, *p* < 0.05

Bold represents there is statistical significance between the groups, *p* < 0.05

## Discussion

The slipped vertebra reduction remains a controversial issue in the surgical treatment of lumbar spondylolisthesis. In 2011, Audat et al. [5] reported that spondylolisthesis reduction is unnecessary as the clinical outcomes remain the same with or without reduction. However, other studies confirmed marked decreases in SD. Sears et al. [6] reported a good clinical outcome in 83% of cases after slip reduction of spondylolisthesis. In a prospective study involving 40 patients with degenerative spondylolisthesis, Wegmann et al. [7] demonstrated that reduction strongly correlates with improvements in quality-of-life scores (QLS). Spondylolisthesis reduction can, therefore, restore spinal canal volume, correct tapering of the nerve root hole, protect nerve roots from being pulled, and regain the overall physical arrangement of the vertebral body. In addition, decrease in SD may have a positive effect on LL correction. Kawakami et al. [8] reported that a decrease in SD was highly associated with enhanced LL correction. If the SD reduction of the slipped vertebra in lumbar spondylolisthesis is successful, compressive techniques with posterior instrumentation can be readily performed. There is no technical difficulty in treating lumbar spondylolisthesis with an additional screw in the proximal vertebral body. In fact, the upper screws can serve as a leverage point and the upper and lower displaced vertebral bodies can act as anchors to facilitate a middle bridge-shaped pulling reduction, under direct vision. Adding a screw to the proximal vertebral body produces a stronger lifting force, a more uniform stress distribution, and an enhanced secure reduction effect. The risk of pedicle screw extraction can also be reduced, particularly in patients with osteoporosis. In this study, SD was significantly decreased in the 6S group, compared to the 4S group at all points after surgery. This indicated that SD was significantly decreased by introducing the additional upper pedicle screws in PLIF surgery. Hence, patients in the 6S group, relative to the 4S group, received sufficient distraction reduction.

Lumbar spondylolisthesis almost always occurs in the lumbosacral segment, which is similar to the thoracolumbar segment, owing to its unique anatomical and biomechanical features. Prior studies reported [9, 10] that about 70% of the global LL are located in the last two lumbar levels. During lumbar spinal fusion surgery, LL recovery is a priority. However, it is difficult to mediate, especially, in terms of the acquisition of total LL during surgery. Takahashi et al. [11] reported that an increased SL in turn increases LL. Therefore, SL acquisition leads to LL. In this study, the postoperative LL and SL of both groups were significantly improved, compared to before surgery. After PLIF surgery, no significant differences were observed in LL and SL values between the two groups, but the LL and SL values in the 6S group were better maintained at the last follow-up. We speculate that this is due to the varying fixation methods used during surgery. The 6 screws in 3 vertebrae were obviously more superior to the 4 screws in 2 vertebrae, particularly, in terms of fixation strength and stress distribution, in the fixation mode of lumbar spondylolisthesis.

The sagittal sacropelvic morphology and orientation intercede lumbar spinal geometry. In case of abnormal sacropelvic morphology and orientation, a disturbed global sagittal spinal balance is achieved. PI is a fixed parameter that is independent of pelvis orientation. PI is equal to the arithmetic sum of SS and PT [12] . The standard PI value is approximately 53° ± 9° [13]. In this study, PI was significantly higher, compared to the standard value, which is coincident with other publications [14]. There was also a close relationship between PI and LL (the ideal relationship is: LL = PI ±9° [15]. Patients with a high PI value require more extended LL after surgery in order to maintain sagittal balance. PT and SS, on the other hand, are positional and are related to pelvic orientation. The standard PT value is approximately 13 ± 6° [16]. Kim et al. [17] reported that PT has a strong correlation with good clinical outcomes. Additionally, PT improvement after PLIF surgery may be one of the reasons for low back pain relief in patients with lumbar spondylolisthesis. In this study, we showed no statistically significant differences in PI and SS values between the two groups. Although there was significant difference between the two groups in PT values at the last follow-up, the difference was small. Therefore, it appears that short and long segment pedicle screw fixations do not massively influence the spinopelvic sagittal plane after surgery. However, the postoperative patients in this study lacked in performing daily activities, mostly due to old age. Meanwhile, the follow-up time was short, and could not accurately reflect the effect of screw numbers on spinal-pelvic sagittal balance.

Compared to the preoperative status in this study, VAS and ODI values decreased significantly after surgery in both groups. Although most patients reported satisfied clinical functional improvement after surgery, three patients in both groups suffered from chronic low back pain. Rajnics et al. [18] reported that failed restoration of LL may cause chronic low back pain post surgery. This is likely due to the painful compensation with hyperextension at the upper adjacent levels, due to the increase in traction load on the posterior spinal arrangement. LL restoration did not change, and even worsened, in these three patients with chronic low back pain in our study.

Lee et al. [19] reported that the incidence of adjacent segment disease (ASD) is markedly higher after PLIF surgery. This is because the spinal fixation instruments and fusion cage can significantly increase segment stiffness and stress transmission to the adjacent segment, thus accelerating the postoperative degeneration process of the adjacent segment. Similarly, some scholars speculate that long segment fixation will promote normal segment degeneration, since the longer lever arm from the multi-segment fusion will generate more stress on the remaining free segment [20]. Gene et al. [21] advocated the new-onset substantial mechanical back pain as a possible symptom of ASD. However, Kumar et al. [22] reported that longer level fusions or a PLIF addition does not increase ASD risk. Till date, studies have not definitively revealed whether the radiological alterations and clinical deterioration of ASD are the result of spinal fusion or iatrogenic production of a rigid motion segment. The exact mechanism of ASD is uncertain, but some risk factors, such as, pre-existing degeneration of the adjacent segment, exert a negative impact on ASD. Moreover, secondary degeneration of the proximal segment of spondylolisthesis may occur due to alterations in the normal spinal sequence after lumbar spondylolisthesis. Lee et al. [23] reported that pre-existing disc degeneration increases biomechanical changes in adjacent segment after surgery. Some surgeons might perform a complete PLIF at the adjacent level. However, Zhang et al. [24] demonstrated that a distraction of the intervertebral space and facet fusions in the adjacent segment can effectively prevent ASD. During PLIF surgery, bone graft fusion is generally performed to correct spondylolisthesis. However, in this study, the normal segment was only fixed without fusion in the 6S group, which achieved augmented LL and SL, compared to the 4S group. In terms of longer segmental fixation, such as, L1-S1 fixation, the L4-S1 fixation is known to avoid the complete loss of lumbar activity, induce less surgical trauma, and reduce the cost of internal fixation. Therefore, in this study, two segment fixation was recommended for patients with isthmic L5–S1 spondylolisthesis, with a long course of disease, degeneration of adjacent segments, and low bone density.

There were certain limitations in our study. Firstly, this was a retrospective study. Hence, our nonrandomized design may have unintentionally introduced selection bias. Secondly, our patient population was relatively small. Finally, the mean follow-up time was relatively short (less than 2 years). In future investigations, we recommend conducting prospective multi-center studies, involving large patient population, and long-term follow up.

## Conclusion

We employed two approaches to examine the post-surgical lumbar spinal sagittal stabilities and clinical functional outcomes of lumbar spondylolisthesis patients. Based on our analysis, both 4S and 6S markedly these parameters after surgery. However, relative to the 4S group, the 6S group exhibited a greater advantage in restoration and maintenance of radiographic parameters.

## Data Availability

The datasets used and/or analyzed during the current study available from the corresponding author on reasonable request.

## Abbreviations

PLIF: Posterior lumbar interbody fusion
BMD: Bone mineral density
BMI: Body mass index
LL: Lumbar lordosis
SL: Segmental lordosis
PI: Pelvic incidence
PT: Pelvic tilt
SS: Sacral slope
SD: Slippage degree
SVA: Sagittal vertical axis
CLBP: Chronic low back pain
VAS: Visual analogue scales
ODI: Oswestry Disability Index
